# The complete chloroplast genome of the threatened Napa False Indigo *Amorpha californica* var. *napensis* Jeps. 1925 (Fabaceae) from Northern California, USA

**DOI:** 10.1080/23802359.2022.2029605

**Published:** 2022-01-27

**Authors:** Hartnell College Genomics Group, Ivan D. Agudelo, Griselda Aldaco, Angel Brito-Pizano, Kimberly G. Chavez, Karina G. Cortina, Jorge Flores, Alejandro Fuentes, Adam N. Garcia, Alejandro Garcia, Daniel Gonzalez-Martinez, Jennifer Hernandez Ramos, Jeffery R. Hughey, Fernando R. Katada, Felix A. Leon, Maleny P. Lopez, Sandra Z. Lopez, Aileen G. Mendoza, Maritta Molina, Asmahan Muhrram, Daisy Ortiz-Matias, Tonantzin E. Ortiz, Alicia Pacheco, Nandini Patel, Paz M. Ramirez, Jennifer L. Scaramuzzino, Alexandria Soto, Richard A. Stabler, Jessica M. Vidauri, Jose Villicana, James A. Yhip

**Affiliations:** a Division of Mathematics, Science, and Engineering, Hartnell College, Salinas, CA, USA; b County of Sonoma, Santa Rosa, CA, USA

**Keywords:** *Amorpha*, *Amorpha californica*, *Amorpha californica* var. *californica*, chloroplast genome, Papilionoideae

## Abstract

*Amorpha californica* var. *napensis* Jeps. 1925, the Napa false indigo, is a threatened shrub endemic to northern California. Here the complete chloroplast genome of topotype material of var. *napensis* was assembled and characterized to contribute to the bioinformatics, systematics, and conservation of this variety. The chloroplast genome (GenBank accession OK274088) is 158,294 base pairs (bp) in length, encodes 130 genes including 85 protein-coding, 37 tRNA, 8 rRNA, and shows a high-level of gene synteny to other Papilionoideae. Phylogenetic analysis fully resolved var. *napensis* in a clade with *A. fruticosa* L. and *A. roemeriana* Scheele, sister to the Dalbergieae. The newly sequenced chloroplast genome shows that the genetic differences between var. *napensis* and *Amorpha californica* Nutt. var. *californica* are greater than the variation observed between var. *napensis* and many other *Amorpha* spp. sequences deposited in GenBank. These data suggest that var. *napensis* should be elevated to full species rank.

*Amorpha californica* var. *napensis* is a deciduous shrub originally described by W.L. Jepson from specimens collected from Moore Creek, Howell Mountain, Napa County, California (Jepson 1925). The variety was said to differ from *A. californica* Nutt. var. *californica* in being subglabrous (vs. minutely pubescent), lacking glands on the rachis (vs. prickle-like glands), having shorter racemes 2.5–3.18 cm in length (vs. 5–14 cm), and displaying minute teeth on the calyx (vs. long teeth) (Jepson 1925). Abrams (1944) considered var. *napensis* a form of *A. californica* var. *hispidula* (Greene) E.J.Palmer, whereas Munz (1959) treated the latter as a synonym of var. *napensis*. Wilbur (1975) accepted the varietal status of var. *napensis*, but placed var. *hispidula* in synonymy under *A. californica*. Hickman (1993) and Baldwin et al. (2012) recognized the variety, but narrowed its distribution to Napa, Marin, and Sonoma counties where it occurs in Chaparral communities less than 800 meters in elevation. Calflora (https://www.calflora.org/) currently lists var. *napensis* as 1B.2 (fairly threatened in California). Based on a survey of this variety from the type locality, it appears nearly extirpated due to vineyard expansion in the famed Howell Mountain American Viticultural Area. To date, the only data deposited in GenBank for var. *napensis* are three barcode sequences determined from a single specimen (Straub and Doyle 2014). We assembled and analyzed the complete plastid genome of topotype material of var. *napensis* to contribute to the bioinformatics, systematics, and conservation efforts of this threatened variety.

The specimen of var. *napensis* analyzed in this study was collected in accordance with guidelines provided by Napa county and Hartnell College from the north end of Moore Creek, Angwin, California (38°33′55.0152″N 122°24′18.8028″W) and deposited in the herbarium at Hartnell College (https://www.hartnell.edu/, Jeffery R. Hughey, jhughey@hartnell.edu) under voucher number HCC 266. The DNA was extracted following the methods outlined in Hughey et al. (2019). The 150 bp PE Illumina library construction and sequencing was performed by Quick Biology (Pasadena, California, USA) and yielded 24,733,870 reads. The adapters and low quality reads were removed using the Trim Adapters and Trim Low Quality default settings with the BBDuk plugin in Geneious Prime version 2019.1.3 (Biomatters Limited, Auckland, New Zealand). The genome was assembled by mapping the reads onto the reference genome *A. fruticosa*, GenBank accession number MN709789 (Zhang, Wang, et al. 2020), using the Medium-Low Sensitivity/Fast setting in Geneious Prime. The gaps were closed by iterative mapping using the same settings in Geneious Prime. The annotation was performed using the default settings in GeSeq (Tillich et al. 2017) and CPGAVAS2 (Shi et al. 2019), followed by adjustments according to NCBI ORFfinder, Sequin 15.5, and tRNAscan-SE 1.21 (Schattner et al. 2005). The var. *napensis* complete chloroplast nucleotide sequence was aligned to 25 other papilionoid and three outgroup taxa from the Dialioideae using the auto settings in MAFFT (Katoh and Standley 2013). The ML phylogenetic analysis was executed with the TVM + F + I + G4 substitution model and 1000 ultrafast bootstrap replicates in W-IQ-TREE (Trifinopoulos et al. 2016). The tree was visualized with TreeDyn 198.3 at Phylogeny.fr (Dereeper et al. 2008).

The complete chloroplast genome of var. *napensis* is 158,294 bp in length and exhibits a standard quadripartite structure (Shinozaki et al. 1986; Wicke et al. 2011). The genome contains an LSC, SSC, and two IRs with lengths 88,110 bp, 18,580 bp and 25,802 bp, respectively. The GC content is 36.0%. Gene content and organization show a high-level of synteny to *A. fruticosa* and *A. roemeriana* (GenBank accession number MW628937) (Zhang, Wang, et al. 2020; Lee et al. 2021). The chloroplast genome of var. *napensis* is 99.86% similar in nucleotide sequence to *A. fruticosa* and 99.87% to *A. roemeriana*. BLAST analysis of the var. *napensis* genome found an identical petN-psbM intergenic spacer sequence and nearly identical trnT-trnD intergenic spacer sequence that differed by 1 bp from var. *napensis* from Angwin, California (Straub and Doyle 2014). In comparison, var. *napensis* differed from var. *californica* from the Santa Rosa Mountains, California in its petN-psbM sequence by 5 bp (99.0%) and trnT-trnD sequence by 12 bp (99.24%). The var. *napensis* sequences were greater in genetic distance to var. *californica* than to many other species classified in *Amorpha*.

Phylogenetic analysis of representative Papilionoideae fully resolved var. *napensis* in a clade with *A. fruticosa* and *A. roemeriana* in a sister position to the Dalbergieae (Figure 1). These results are consistent with the evolutionary relationships inferred for the Amorpheae based on multigene analysis (McMahon and Hufford 2004), *mat*K sequencing (Cardoso et al. 2012, 2013), and a schematic compilation based on other published works (The Legume Phylogeny Working Group 2013). Given the high degree of plastid marker sequence variation between var. *napensis* and var. *californica,* phylogenetic analysis of barcode markers or the chloroplast genome of var. *californica* from its type locality in Santa Barbara, California, are necessary to test the hypothesis that var. *napensis* should be recognized at the species level.

**Figure 1.**
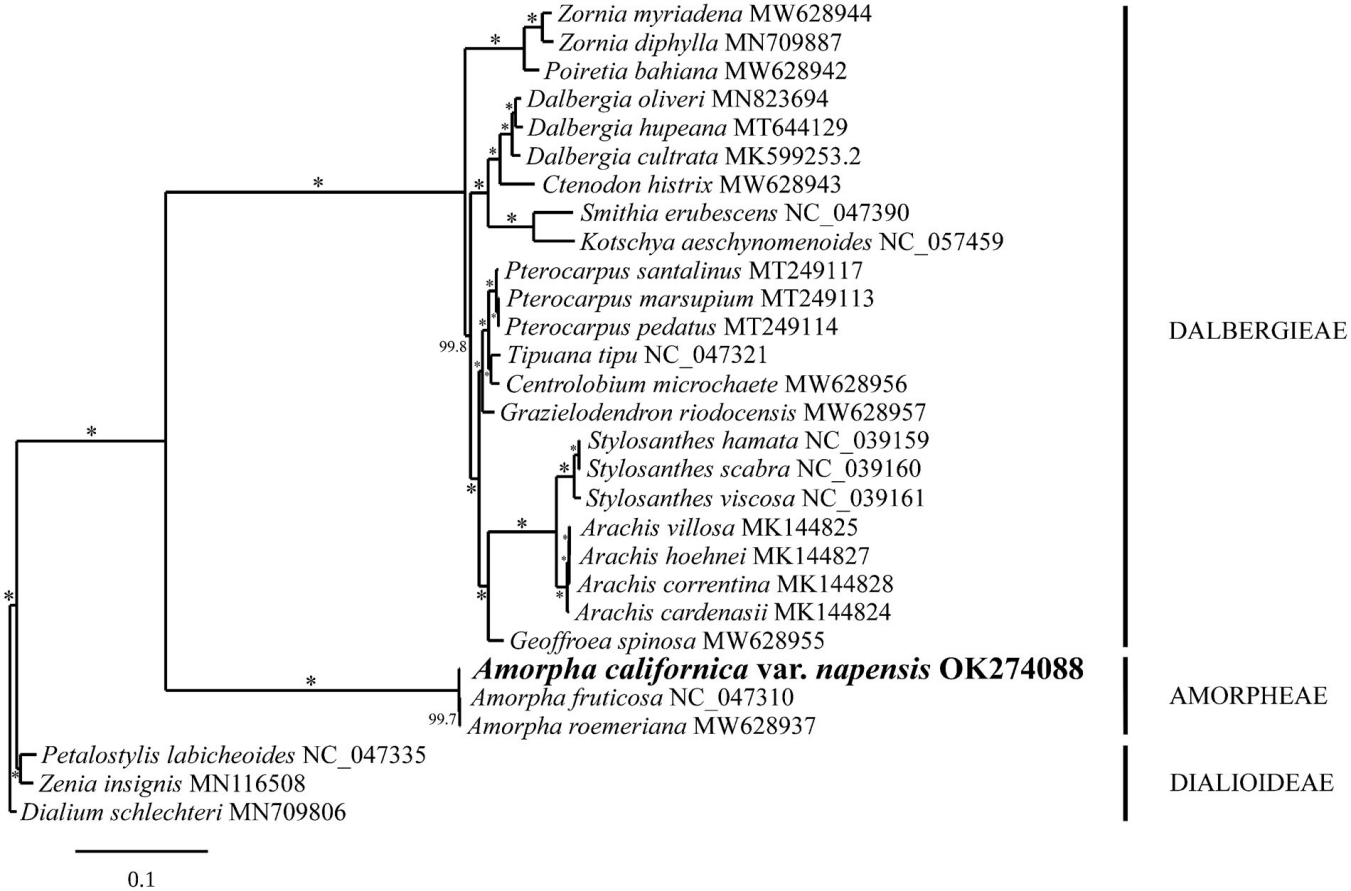
RaxML phylogram of the complete chloroplast genome of *Amorpha californica* var. *napensis* and related Papilionoideae. Numbers along the branches are ML bootstrap supports based on 1000 replicates (* indicates 100% boostrap support). The legend below represents the scale for nucleotide substitutions. The following accessions with references were used for the phylogenetic anlaysis: *Amorpha roemeriana* MW628937*, Centrolobium microchaete* MW628956, *Ctenodon histrix* MW628943, *Geoffroea spinosa* MW628955, *Grazielodendron riodocensis* MW628957, *Poiretia bahiana* MW628942, *Zornia myriadena* MW628944 (Lee et al. 2021); *Amorpha fruticosa* NC_047310, *Dialium schlechteri* MN709806, *Petalostylis labicheoides* NC_047335, *Smithia erubescens* NC_047390, *Tipuana tipu* NC_047321, *Zornia diphylla* MN709887 (Zhang, Wang, et al. 2020); *Pterocarpus marsupium* MT249113, *Pterocarpus pedatus* MT249114, *Pterocarpus santalinus* MT249117 (Hong et al. 2020); *Stylosanthes hamata* NC_039159, *Stylosanthes scabra* NC_039160, *Stylosanthes viscosa* NC_039161 (Marques et al. 2018); *Arachis cardenasii* MK144824, *Arachis hoehnei* MK144827, *Arachis villosa* MK144825 (Wang et al. 2019); *Dalbergia hupeana* MT644129 (Hong et al. 2021); *Dalbergia oliveri* MN823694 (Zhang, Li, et al. 2020); *Kotschya aeschynomenoides* NC_057459 (Oyebanji et al. 2020); *Dalbergia cultrata* MK599253 (Liu et al. 2019); *Zenia insignis* MN116508 (Lai et al. 2019); *Amorpha californica* var. *napensis* OK274088 (this study).

## Data Availability

The genome sequence data that support the findings of this study are openly available in GenBank at (https://www.ncbi.nlm.nih.gov/) under the accession number OK274088. The associated BioProject, SRA, and BioSample numbers are PRJNA765780, SRR16037173, and SAMN21583931 respectively.
